# The Functional Interaction Between Epstein–Barr Virus and MYC in the Pathogenesis of Burkitt Lymphoma

**DOI:** 10.3390/cancers16244212

**Published:** 2024-12-18

**Authors:** Sandra Solares, Javier León, Lucía García-Gutiérrez

**Affiliations:** Instituto de Biomedicina y Biotecnología de Cantabria, Departamento de Biología Molecular, Universidad de Cantabria-CSIC, Albert Einstein 22, 39011 Cantabria, Spain; sandra.solares@alumnos.unican.es (S.S.); javier.leon@unican.es (J.L.)

**Keywords:** MYC, Epstein–Barr virus, Burkitt lymphoma

## Abstract

The Epstein–Barr virus (EBV) is associated with a wide range of diseases, malignant and non-malignant. EBV was, in fact, the first virus described with cell transformation capacity, discovered by Epstein in 1964 in lymphoma samples from African children. Since then, EBV has been associated with several human tumors including nasopharyngeal carcinoma, gastric carcinoma, T-cell lymphoma, Hodgkin lymphoma, diffuse large B cell lymphoma, and Burkitt lymphoma among others. The molecular hallmark of Burkitt lymphoma (BL) is a chromosomal translocation that involves the *MYC* gene and immunoglobulin loci, resulting in the deregulated expression of *MYC*, an oncogenic transcription factor that appears deregulated in about half of human tumors. The role of MYC in lymphoma is well established, as MYC overexpression drives B cell proliferation through multiple mechanisms, foremost, the stimulation of the cell cycle. Indeed, MYC is found overexpressed or deregulated in several non-Hodgkin lymphomas. Most endemic and many sporadic BLs are associated with EBV infection. While some mechanisms by which EBV can contribute to BL have been reported, the mechanism that links *MYC* translocation and EBV infection in BL is still under debate. Here, we review the main EBV-associated diseases, with a special focus on BL, and we discuss the interaction of EBV and *MYC* translocation during B cell malignant transformation in BL.

## Simple Summary

The Epstein–Barr virus (EBV) infection does not induce any apparent pathology in most people but it has been associated with an increased risk of developing a number of non-malignant diseases (e.g., infectious mononucleosis and multiple sclerosis) and some cancers. Among these, the association between EBV and Burkitt lymphoma (BL) is striking, involving a tumor where *MYC* is deregulated by translocation in all cases. BL is more prevalent in children from equatorial Africa (>90% of the cases) whereas the association of EBV with BL is much lower (25–40%) in other regions. This high association suggests that EBV is a driving mechanism, but whether it is sufficient to trigger lymphomagenesis or it is a cooperative factor is under debate. Indeed, the precise molecular mechanisms underlying the virus activity in infected B cells in collaboration with MYC is still unclear. The molecular mechanisms by which EBV operates in tumor B cells will be discussed.

## 1. The Epstein Barr Virus, General Introduction, and Incidence

The Epstein–Barr virus (EBV) is a human herpesvirus (known as Human Herpesvirus 4 or HHV-4) that belongs to the gamma-herpesvirus subfamily. It was identified by Epstein in 1964 in samples of Burkitt lymphoma (BL) from African children [1]. The majority of higher primates have their own EBV-like virus, but humans serve as the only natural host for EBV. Two major types of EBV have been reported: EBV1 and EBV2, also known as A and B, respectively, being the sequence of the genes that encode for their nuclear antigens considering the main difference between them [2]. While EBV is detected worldwide, EBV1 and EBV2 seem to have different prevalences depending on the geographical location [3,4]. EBV1 is the most frequent subtype globally. However, in specific regions such as Central Africa, Papua New Guinea, or Alaska, EBV2 is the most prevalent one. In the rest of the world, EBV2 is very rare and only found in immunosuppressed individuals [3].

EBV infection is associated with a wide range of diseases, either malignant or non-malignant, which can be classified into (i) non-malignant: infectious mononucleosis and autoimmune diseases (multiple sclerosis, rheumatoid arthritis, and lupus erythematosus); (ii) non-lymphoid cancers: nasopharyngeal carcinoma, gastric cancer, and lymphoepithelial-like cancer; and (iii) lymphomas: BL, T cells and NK lymphoma, Hodgkin lymphoma, DLBCL, and lymphomas of immunosuppressed individuals. The incidence of EBV infection is very high. More than 90% of the human population become infected at some point in their lives with EBV, a fact that was already reported during the 70s and continues to be reported [5,6]. Thus, EBV is likely the most ubiquitous virus infecting humans. EBV is mainly spread from saliva-containing virus-infected epithelial cells, but it can be also transmitted through blood transfusions or organ transplants [7]. The primary contact with EBV usually happens during childhood and its first and most common form of presentation is infectious mononucleosis [8]. However, it is important to highlight that, despite EBV prevalence, only a low percentage of people among the infected population develop any EBV-associated disease, as discussed later.

## 2. EBV Genome and Latency Programs

**Figure 1:**
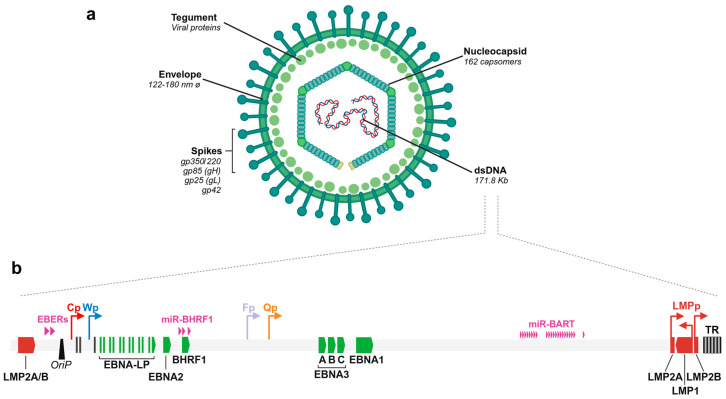
Epstein–Barr virus (EBV) structure and genome. (**a**) Scheme depicting the most relevant features of the EBV structure. *Ø*: virion diameter. (**b**) Schematic representation of the EBV linearized genome. Green and red boxes represent the EBNAs and LMPs latent genes, respectively. Violet arrows represent ncRNAs. The four alternative promoters from which EBNA genes are transcribed are represented by colored arrows (Cp, Wp, Fp, Qp). *OriP*: Replication origin. TR: Terminal Repeats. Created in BioRender. Garcia, L. (2024) BioRender.com/y04j261.

The EBV is a 122–180 nm diameter virus and consists of a DNA core inside a 162 capsomers-nucleocapsid surrounded by an envelope. The inner part between the nucleocapsid and the envelope is composed of a protein viral tegument, while the outer envelope carries the external virus-encoded glycoprotein spikes [9]. Among these, gp350/220 is the most abundant glycoprotein, together with gH, gL, and gp42, which comprise the machinery that mediate the virus entry into the host cell [10] (Figure 1a).

The EBV genome consists of a linear double-stranded DNA of 171.8 Kb in length, which encodes for more than 94 protein-coding genes (NCBI Reference Sequence: NC_007605.1) (Figure 1b). The nomenclature of the open reading frames (ORFs) is determined by their location with respect to the BamHI fragment. These ORFs are grouped into latent or lytic, and the lytic group is also subdivided into immediate early, early, and late. EBV also encodes non-coding RNAs (ncRNAs), including EBV-encoded RNAs or EBERs [11] and 44 mature miRNAs [12] (from 25 precursor miRNAs, according to https://www.mirbase.org/ accessed on 12/08/2024). The miRNAs are grouped into two clusters, depending on whether they are encoded within the BHRF1 or BART regions, known as ebv-miR-BHRF1 or ebv-miR-BART, respectively. The viral genome also contains 0.5 Kb terminal repeat sequences (TRs) located either at both termini [13,14] or within internal regions, which divide the genome into short and long unique sequences [15,16]. The repeats at the terminal regions serve as good markers to identify if specific EBV-infected cells derive from a common progenitor.

**Figure 2:**
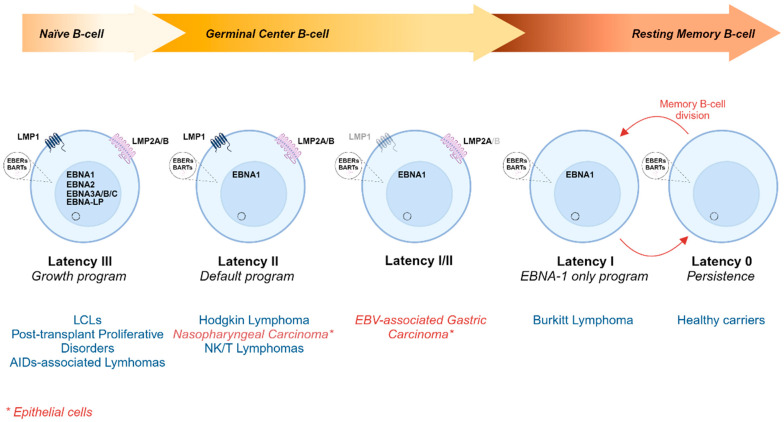
EBV latency programs and associated malignancies. After primary infection, the infected cell enters the Latency III program, also known as the growth program, expressing all latent genes, BARTs, and EBERs. This activates B cells promoting their transition toward a germinal center reaction and switching to Latency II or the default program, with a more restricted gene expression pattern. Finally, memory B cells exit the germinal center showing the Latency 0 program, also known as “Persistence”, in which only ncRNAs are expressed. Eventually, the transition from Latency 0 to Latency I (or the EBNA-1 only program) may occur when memory B cells proliferate so that the EBV episome is properly segregated to the daughter cells. In blue, EBV-associated lymphomas with their corresponding latency program. In red, EBV-associated epithelial malignancies and their corresponding latency programs are included. Created in BioRender. Garcia, L. (2024) BioRender.com/y04j261.

EBV has the ability to infect B cells, transforming them into Lymphoblastoid Cell Lines (LCLs) in vitro. After the primary infection in vivo, the virus silences almost completely its genome to avoid the host immune surveillance, allowing the establishment of a persistent infection. Thus, EBV is found in a dormant state or latent infection for most of its life within the host. This latency can be classified into four different programs (0, I, II, III) depending on the expression of the EBV latency genes (reviewed in [17,18,19]). Interestingly, each EBV-associated cancer shows one specific EBV latency program, suggesting an important role of the different latency genes in promoting particular tumors (Figure 2). The different transcriptional program activated is based on complex epigenetic regulations selecting viral promoters (reviewed in [20]). Genes expressed along the different latency programs are transcribed from specific promoters within the EBV genome (Figure 1b). In latency 0 (also known as the germinal center model of EBV persistence), only EBERs and miRNAs are expressed. Latency I is characterized by the expression of EBNA1 along with EBERs and BamH1A transcripts, and it is characteristic of BL. In latency II (or the default program), EBNA1, LMP1, LMP2A/B, EBERs, and BamH1A transcripts are expressed and are typically found in nasopharyngeal carcinoma and Hodgkin lymphoma. The promoter usage in latency 0, I, and II is the same, given that EBNA1 is transcribed from the Qp promoter, while Wp and Cp are silenced [21,22,23]. Latency III (also known as growth program) expresses all latent proteins: six EBNAs (EBNA1, EBNA2, EBNA3A/B/C, and EBNA-LP) and three LMPs (LMP1, LMP2A/B), together with EBER 1 and 2 and BARTs. In this case, promoters Wp and Cp drive the expression of all EBNA genes [24,25]. This type of latency is found in LCLs and in the majority of post-transplant lymphoproliferative disorders [26]. The model of EBV persistent infection states that EBV uses its growth program to activate naïve B cells, forcing them to undergo a germinal center reaction, in the absence of a foreign antigen, leading to a long-lived memory B cell in which EBV latently persists (reviewed in [27,28]). 

The latent state of the EBV is concurrent with the establishment of a persistent infection as a mechanism to avoid host immunity, with the limited expression of specific gene sets. However, upon still poorly understood mechanisms, a switch from the latent infection to the lytic replication may occur, leading to the formation of new virion particles. While this is mainly characteristic of epithelial cells and salivary glands, allowing the spread of viral particles toward new cells [29,30], B cells also undergo the EBV lytic replication cycle. This switch from EBV latent to lytic cycle has been related to the cell differentiation processes of both epithelial and B cells [27]. The lytic cycle is divided into three sequential phases, which also classify the gene sets expressed within each of them: (i) immediate-early genes: mainly transcription factors in charge of triggering the lytic cascade [31,32,33]. *BZLF1* and *BRLF1* are the two determining immediate-early genes, simultaneously expressed and needed for the transition from latent to lytic infection [34]; (ii) early genes: genes encoding nucleotide metabolism-related enzymes (dispensable due to redundancy with host’s enzymes), the vPIC (viral preinitiation complex) composed of BcRF1 and another five cofactors that interact with the cellular RNA pol II [35], and DNA replication proteins, essential for the proper replication of the viral DNA and the early to late phase transition [36]; and (iii) late genes: structural proteins for the assembly of the new virions, allowing the packaging of the viral DNA and further release and spread of the new particles from productively infected cells. This process has been extensively reviewed elsewhere [37].

## 3. EBV Receptors in Human Cells and Infection

Depending on the cell type, different viral surface glycoproteins are involved in the recognition and interaction with the host cell, leading to different EBV mechanisms of entrance in the cell. In the case of B cells, this process has been extensively studied and is very well known. However, the mechanism through which EBV interacts and enters epithelial cells is much more poorly understood, although several molecules and processes have already been reported. The EBV glycoproteins gB and the gH/gL complex are needed for the virus membrane fusion with both B cells and epithelial cells [38]. gB activates the viral membrane fusion with that of the host cell [39], and gH/gL regulates this fusion process [40]. While in B cells EBV entrance takes place through endocytosis followed by the fusion of the viral membrane with the endocytic vesicle membrane of the cell [41], it has been described that in epithelial cells, EBV can either enter the cell by direct fusion of the virus membrane with the cell membrane or via lipid rafts endocytosis or micropinocytosis [42].

The glycoprotein CR2 (Complement Receptor 2, also called CD21) is the EBV-cell receptor in B cells [43,44]. CR2 is expressed in mature B lymphocytes and B cell lines but not in early pre- and pro-B cells. It is also found in peripheral blood and thymic T cells and T cell lines [45]. CR1 (Complement Receptor 1, CD35) has also been reported to serve as an EBV receptor [46], but so far, the information on CR1 as an EBV receptor is limited.

CR2 recognizes specific C3d fragments and acts as a co-receptor for B cell receptors (BCR). It is found in a complex with CD19 or CD81 [47]. CR2 is also expressed in follicular dendritic cells [48]. The importance of the CD19-CR2 complex has been demonstrated in vivo as mice lacking either CD19 or CR2 showed reduced formation of germinal center and primary antibody responses [49,50,51,52]

CR2 plays an important role in enhancing the immune response promoting cell proliferation in preactivated B cells. In follicular dendritic cells, CR2 rescues antigen-activated B cells from apoptosis and promotes somatic hypermutations and class switch recombination [51]. CR2 has a very short intracellular domain, suggesting that it is very unlikely that it may act as a signal transducer. However, CR2 interaction with CD19 in close proximity with BCR enhances BCR activation and its downstream signaling [45]. 

**Figure 3:**
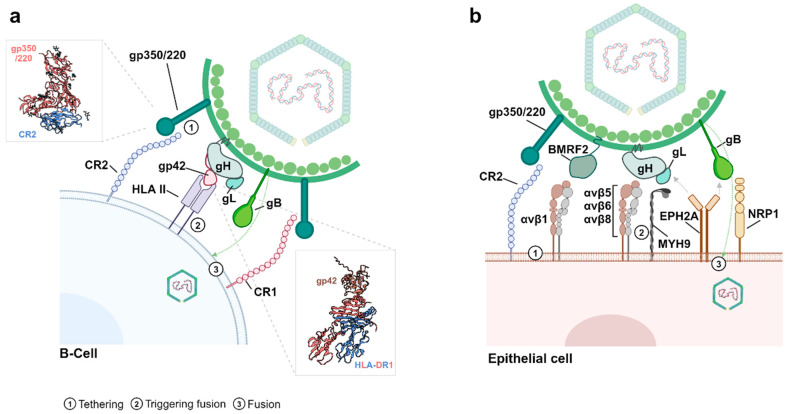
Molecules implicated in the interaction and entry of EBV with its host cell. (**a**) Scheme depicting EBV interaction with B cells. gp350/220 from the EBV envelope interacts with CR2 (or CR1), facilitating gp42 interaction with the HLA class II of the B cell surface. This triggers EBV fusion with the B cell membrane through gB to further deliver the virus genoma inside the cell. PDB: 8SM0 (structure of gp350/220 in complex with CR2). PDB: 1KG0 (Epstein–Barr virus gp42 bound to the MHC class II Receptor HLA-DR1). (**b**) Scheme depicting the EBV interaction with epithelial cells. gp350/220 or BMRF2 recognizing CR2 or β1-integrins, respectively. gH/gL interact with different β5, β6, and β8 integrins and MYH9 in the cell surface. gB, which drives the fusion with the cell membrane, interacts with NRP1. EPH2A binds both gH/gL and gB. Created in BioRender. Garcia, L. (2024) BioRender.com/y04j261.

The binding of EBV to B cells takes place through the EBV envelope glycoprotein gp350/220 interaction with CR2 [44,53,54], promoting the viral entrance into the host cell by endocytosis [55]. The viral envelope fuses with the cell membrane through a mechanism involving three other viral proteins: gp85 (gH), gp25 (gL), and gp42 [10,38,56]. Since gp42 also binds to the HLA class II, the virus uses this complex as a cofactor to infect B-lymphocytes [56,57,58] (Figure 3a). Although EBV is considered a B-lymphotropic virus, other cell types such as T lymphocytes and epithelial cells can be infected by EBV, since the virus has been detected in different T lymphomas, nasopharyngeal carcinomas, and gastric cancer, among others. Therefore, EBV can infect cells independently of CR2 [59], although with lower efficiency.

In the case of epithelial cells, the EBV can interact with the cell surface through gp350/220 when these cells express CR2 [60]. For epithelial cells that do not express CR2, different receptors have been described so far, including integrins, neuropilin-1 (NRP-1), non-muscle myosin heavy chain IIA (NMHC-IIA, also called MYH9), and EPHA2 (Figure 3b). Integrins αvβ5, αvβ6, and αvβ8 interact with EBV through gH/gL [61,62]. Also, integrin β1 is able to interact with BMRF-2, facilitating EBV attachment to epithelial cells. NRP-1 is a neuropilin able to interact with EBV gB in epithelial cells, promoting viral entrance into the host cell [42]. NMHC-IIA (MYH9) was described as another EBV receptor in nasopharyngeal carcinoma, which interacts with gH/gL of the EBV envelope [63]. Ephrin Receptor A2 (EPHA2) was the latest EBV receptor reported in epithelial cells. EPHA2 is a member of the RKT protein family and is known to serve as the entry molecule for KSHV (another human herpesvirus) and other pathogens, apart from EBV [64,65].

Whether EBV primary infection takes place through epithelial cells or B cells is a matter of debate. Different models have been reported, claiming that either B cells, epithelial cells, or both concomitantly are the first cells infected by EBV [66]. However, the most solid evidence suggests that the B cells in the submucosal secondary lymphoid tissues (such as tonsils) are the first contact with the EBV. This is based mainly on two proven facts: (1) polarized epithelial cells from the oral cavity are only infected through their apical surface by direct contact with EBV^+^ lymphocytes [67]; (2) the presence of EBV is detected much earlier in the blood than in epithelial cells from the oral cavity [66]. In fact, the main reservoir of EBV-infected cells is the lymphatic tissue. Upon first contact with the virus after the eventual activation of EBV^+^ lymphocytes, oropharynx cells undergo a lytic replication cycle. This lytic infection leads to the production of new viral particles which are spread through the throat. EBV particles infect resting B lymphocytes from the oropharynx lymphatic tissue, which undergo a growth-transforming (Latency III) infection, promoting the proliferation of B cells and activating the host immune response. B cells migrate toward the follicle, switching from latency III to Latency II, where they initiate the germinal center reaction. Latency 0 memory B cells exit the germinal center establishing the long persistent infection, having very restricted expression of viral genes to avoid the immune surveillance. Eventually, memory B cells can divide. In this case, the cells switch to Latency I, allowing the expression of EBNA1 for EBV replication and proper segregation to daughter cells. Occasionally, memory B cells undergo plasma cell differentiation in the tonsil, which leads to the activation of the lytic replication cycle, releasing the virus to the saliva for its spreading to new host individuals or the infection of new epithelial cells or B cells.

## 4. Non-Malignant EBV-Associated Diseases

### 4.1. Infectious Mononucleosis

Primary infection with EBV during childhood is usually asymptomatic or associated with mild hepatitis. When this primary infection happens during adolescence it can be asymptomatic or associated with acute infectious mononucleosis (IM) [68]. Contact with the virus takes place through saliva. Infants under 2 years old infected with the EBV seroconvert asymptomatically, showing activated EBV-specific T cells and viral loads similar to those from adolescents that develop IM but lack lymphocytosis [69]. A racial disparity has been proposed, suggesting that black young children are more susceptible to EBV infection, most likely because of genetic reasons that are still unknown [70,71].

IM appears as a result of the immunological response triggered by EBV infection. Upon EBV-induced B cell proliferation, NK-cell and T-cell responses are activated [66,72,73]. NK-cell population expands in frequency and number showing differences among distinct NK subsets and their anatomical localizations. While there is little or no increase in CD4+ T cell response, the CD8+ T cell population expands concomitantly to the development of symptoms, leading to an increase in the number of mononucleated cells in the blood [66,74]. These are atypical T-cells that characterize IM, known as “Downey cells” [75]. IM is considered an immunopathological disease due to the high secretion of proinflammatory cytokines by the CD8+ T cells. This leads to the most common symptoms observed in IM patients, which include sore throat, fever, abdominal discomfort, and fatigue, due to swollen lymph glands and enlarged spleen and liver [76].

### 4.2. Multiple Sclerosis

Multiple sclerosis (MS) is a chronic inflammatory disease associated with neurodegeneration and central nervous system inflammation due to an altered T-cell response [77]. Among others, EBV is considered a major environmental risk factor for MS development [78,79,80]. Virtually all MS cases are positive for EBV infection and high titers of IgG anti-EBNA-1 before the onset of MS [81,82]. Because of this, EBV has been suggested as a prerequisite for MS. In fact, there is a direct correlation between IM and the risk of developing MS [81]. Also, the combination of EBV with other MS risk factors (such as the presence of the HLA allele *DRB15*) enhances the probability of developing MS [83,84,85]. Indeed, MS patients show elevated titers against EBV years before developing MS symptoms [86].

Whether EBV reaches the brain is unclear. While EBV DNA has been found postmortem in the brain of MS patients in a limited number of studies [87,88,89], other reports have not been able to reproduce those findings lacking convincing evidence of the presence of EBV DNA or infected B cells within the brain, central nervous system, or the cerebrospinal fluid [87,90]. Thus, those theories regarding the mechanism of action by which EBV causes MS that rely on the presence of the EBV within the central nervous system cannot be accepted until its presence within those tissues is demonstrated. One of the most accepted hypotheses by which EBV triggers MS is molecular mimicry. Molecular mimicry refers to the fact that pathogen antigens (EBV in this case) are very similar to specific proteins of the host, leading to an immune response that cannot distinguish between the pathogen and host. This is the case of myelin basic protein (MBP) which, due to its similarity with EBV antigens, is recognized and attacked by T cells from MS patients [91,92,93,94]. Antibodies against EBNA-1 and BFRF3 have been reported to cross-react with HNRNPL and Septin-9, respectively [95,96].

### 4.3. Rheumatoid Arthritis and Systemic Lupus Erythematosus

Besides MS, the infection with EBV is associated with two other autoimmune diseases: rheumatoid arthritis and systemic lupus erythematosus. The possible association of EBV with rheumatoid arthritis (RA) was originally suggested when high titers of anti-EBV antibodies were found in the sera and synovial liquid of RA patients (reviewed in [96,97]). It has been hypothesized that the mechanism can be related to molecular mimicry, as the EBV protein gp110 shares an amino acid sequence with a protein of the DRB1 genes, a Major Histocompatibility Complex (MHC) class II family, the HLA-DRB1*04:01 allele. This epitope would be responsible for raising antibodies in EBV-infected patients [96,98].

The other autoimmune disease associated with EBV is systemic lupus erythematosus (SLR). These patients have autoantibodies against host nuclear antigens and suffer fatigue, systemic pain, skin rash, inflammation, and vascular lesions. Several viruses (e.g., cytomegalovirus, herpesvirus) are known to trigger an autoimmune response, an effect that may also be elicited by EBV [99]. EBV antigens exhibit structural molecular mimicry with common SLE antigens and functional molecular mimicry with critical immune-regulatory components. SLE patients, from a number of unique geographic regions, are shown to have higher rates of EBV seroconversion, especially against early EBV antigens, suggesting frequent viral reactivation. SLE patients also have an increased EBV viral load [96]. The similarity between EBNA1 and at least three SLE autoantigens (SmB/B’, SmD, and 60kdRo) may explain the association of EBV with SLE [100].

## 5. EBV-Associated Carcinogenesis

EBV is the first human oncogenic virus discovered and it has been classified as class I carcinogen by the WHO. It is estimated that EBV is associated with 1–2% of human cancers and 200,000 newly diagnosed cancers each year [101]. Among the different types of cancer associated with EBV, we can divide them into two main groups: non-lymphoma (epithelial cancers) and lymphomas. Non-lymphoma cancers include nasopharyngeal (NPC), lymphoepithelial-like (LELCs), and gastric (GC) carcinomas. Among the lymphomas associated with EBV, we highlight the following: T cell and NK cell lymphoma (TNKL), Hodgkin lymphoma (HL), diffuse large B cell lymphoma (DLBCL), immunosuppressed individual associated lymphomas, and BL. The latter will be discussed separately since it is the main focus of this review.

### 5.1. EBV-Associated Non-Lymphoid Cancers

#### 5.1.1. Nasopharyngeal Carcinoma

Nasopharyngeal carcinoma (NPC) arises from the mucosal epithelium of the nasopharynx. NPCs are divided into three groups according to the latest WHO classification of Head and Neck Tumors: (I) keratinizing squamous cell carcinoma; (II) non-keratinizing differentiated carcinoma; and (III) non-keratinizing undifferentiated carcinoma [102]. Both types II and III (non-keratinizing carcinomas) are associated with high titers of EBV, while type I is not [103,104]. Similarly to BL, the association of NPC with EBV infection is more prevalent in specific geographical areas. In eastern and south-eastern Asia and some areas of the Middle East, where NPC incidence is very high, EBV is associated with >95% of the NPC cases, compared with areas where the NPC incidence is lower, in which EBV association is reduced to ~75% of the cases [104]. Higher incidence and worse prognosis of NPC have been reported for men compared to women [105].

NPC has been suggested to originate from the clonal expansion of a single EBV-infected epithelial cell in the nasopharynx [103]. Thus, the EBV infection occurs prior to the tumor development, which may be promoted by the EBV itself. Before the EBV infection, there is evidence of specific genetic events taking place, which may lead to predisposition to viral infection [106,107]. Healthy nasopharyngeal tissue studies and results from pre-malign tissue biopsies have revealed LOH in chromosomes 3p and 9p and hypermethylation of *RASSF1A* and *CDKN2A* tumor suppressors among other alterations, which may increase EBV infection predisposition (reviewed [108,109]). NPC express the Latency program II with the expression of EBNA-1, LMP-1, LMP-2A/B, EBERs, and BARTs. LMP-1 plays a key role in the regulation of transcription of genes that lead to NPC [110]. However, LMP1 protein expression has been detected only in 2/3 of the cases [111], while in in situ precancerous lesions, its expression has been always observed [103]. This suggests that LMP-1 may be essential during the early stages of NPC development. On the other hand, the intermittent reactivation of the lytic cycle in NPC is associated with tumor progression and immune cell infiltration [112].

Lymphoepithelial-like carcinomas (LELCs) are undifferentiated squamous cell carcinomas with lymphoplasmacytic infiltration of the stroma. LELCs are very similar to nonkeratinizing undifferentiated NPCs and arise at locations other than the nasopharynx. Their association with EBV is not uniform along the different LELCs subtypes, given that the association with EBV is more common in the LELC of the salivary glands, which is the most common LELC. EBV-associated salivary glands LELCs are found in endemic regions (southeast Asian and Arctic Inuit populations), with nearly 100% association, while in non-endemic areas, association with EBV is rarely found [113,114,115]. Sinonasal tract LELCs are also associated with EBV [116], while in other LELC types such as oropharyngeal and laryngeal, EBV association is rare [117,118,119,120].

#### 5.1.2. Gastric Cancer

Gastric cancer (GC) is the fifth cancer most frequently diagnosed worldwide with more than one million new cases every year [121]. Different risk factors have been associated with GC development, including EBV infection [122]. In 1990, Burke and colleagues demonstrated, for the first time, the presence of EBV DNA in gastric carcinoma cells [123]. EBV-associated GC (EBVaGC) constitutes itself as a unique molecular subtype, completely different from EBV-non-associated GC (EBVnGC) [124], and comprises 10% of all GC [125,126]. GC has been classified into four major subtypes: EBV-associated, microsatellite instability, genomic stability, and chromosomal instability [127]. 

There are two theories regarding the primary contact of EBV with gastric epithelial cells: by swallowing saliva containing EBV from the lytic replication of oropharyngeal epithelial cells or by contact with EBV productive infected B cells circulating through the digestive system [128]. It is known that EBV infection of gastric epithelial cells takes place prior to cell transformation, and it is believed that EBVaGC arises from the clonal expansion of a single infected cell [129]. EBVaGC and EBVnGC are very different since each of them develops in anatomically different locations. EBVaGC shows much higher levels of tumor-infiltrating lymphocytes (TILs) (which makes it “immune hot” vs. “immune cold” EBVnGC), cystic gastritis is associated with EBVaGC, and EBVaGC has a better prognosis and overall survival [130]. The viral expression in EBVaGC consists of a Latency I/II intermediate state. EBNA-1, BART, and EBERs are constitutively expressed, while most of them have none or low LMP1 and LMP2B levels and 40% show LMP2A expression [131,132]. Among the genetic alterations and mutations of EBVaGC, *CDKN2A* is found silenced by promoter hypermethylation while MLHI is expressed (opposite to EBVnGC); amplifications of 9p24.1 have been reported leading to gains of *JAK2*, *PD-L1*, and *PD-L2* genes and mutations in PIK3CA and ARID1A have also been found (reviewed in [124,130,133]). 

### 5.2. EBV-Associated Lymphomas

#### 5.2.1. T Cell and NK Cell LYMPHOMA

Although EBV preferentially infects B-lymphocytes, the discovery of EBV DNA in malignant T cells of a rare disease within the nasal cavity broadened the spectrum EBV target cells [134]. EBV-2 is more effective than EBV-1 in infecting T-cells, opposite to what is described for B cells [135]. How EBV infects T lymphocytes is not fully understood yet but it has been described that EBV enters via CR2 [136].

Extranodal NK/T-cell lymphoma, nasal type (ENKTL-NT), is universally associated with EBV, which indicates that the virus may play an important role in the pathogenesis of this disease [134]. ENKTL-NT shows higher incidence in eastern Asia and Latin America compared to Western countries and it represents 1–2% of all NK/T-cell lymphomas [137,138]. ENKTL-NT shows EBV latency I/II, with the expression of EBNA1, LMP2, and variable LMP1. The exact mechanism of how EBV promotes ENKTL-NT is not known. EBV can integrate within specific genomic regions, including an intronic region of the NHEJ1 gene, which has been associated with genome instability [139]. Also, it is believed that the EBV latency program modulates cell signaling processes and prevents apoptosis [140]. Among the molecular signatures of ENKTL-NT, MYC, and NF-κB have been found activated, together with p53 alterations [141].

#### 5.2.2. Hodgkin Lymphoma

Hodgkin lymphoma (HL) consists of a group of diseases arising from transformed B-lymphocytes that accumulate mainly within the lymph nodes, although extranodal disease may also occur. There are two major types of HL, which depend on the type of B cell that becomes transformed: classical HL (cHL), which consists of Hodgkin/Reed–Sternberg (HRS) cells, and nodular lymphocyte predominant HL (NLPHL), in which malignant cells are lymphocyte predominant (LP) [142].

EBV infection is associated with cHL but not with NLPHL, and it is detected in 20–50% of cHL [143]. Several risk factors contribute to EBV^+^ cHL, including HIV infection, clinic history of IM, and the HLA-A*01 allele [144]. Monoclonal EBV genomes have been detected in HRS cells, suggesting an important role of EBV in cHL onset [23,145]. EBV^+^ HRS cells express the Latency II program: EBNA1, LMP1, LMP2A, EBERs, and BARTs (See Section 2; Figure 2). HRS cells show evidence of somatic hypermutation; therefore, they most likely derive from B cells, which have undergone germinal center reactions [146,147]. However, they lack BCR function, which would lead to apoptosis in these cells. The acquisition of anti-apoptotic alterations allows them to survive and contributes to tumor development [144]. EBV infection facilitates the survival of HRS cells. In fact, LMP2A mimics BCR, promoting B cell development, even in the absence of BCR function. In general, EBV^+^ cHL presents a reduced number of chromosomal alterations and aneuploidies when compared to EBV^−^ cHL, which suggests that the EBV latency genes promote the deregulation of the signaling pathways needed for malignant transformation (reviewed in [148]).

#### 5.2.3. Diffuse Large B Cell Lymphoma

Diffuse large B cell lymphoma (DLBCL) is the non-Hodgkin lymphoma most frequently diagnosed (25–35% of all lymphoma cases) [142]. Between 5 to 15% of the DLBCL are EBV-infected and correlate with a poorer prognosis than EBV-negative DLBCL. While in Western countries, EBV^+^ DLBCL is less frequent, the highest percentages of EBV-associated DLBCL are found in Asian and South American countries [149]. In general, older people (>50 years old) suffer an extranodal disease, while younger patients show a nodal disease. 

EBV-infected DLBCL usually expresses either the latency II or the latency III programs [150]. This might reflect the potential dual theory supporting that EBV can infect both naïve B cells and memory B cells. EBV^+^ DLBCL appears to have a lower dependency on cellular events since EBV genes likely contribute to carcinogenesis. EBV^+^ is considered an independent DLBCL entity due to the extensive molecular differences with other subgroups of this disease [151].

#### 5.2.4. Immunosuppressed Individual-Associated Lymphomas

Individuals who suffer immunodeficiencies are prone to develop EBV-related lymphoproliferative disorders (LPDs). Among these, the most common cases are those patients with AIDS or individuals who are receiving immunosuppressive therapies due to organ transplants. The latter, post-transplant lymphoproliferative disorders (PTLDs), are typical after both solid organ and hematopoietic stem cell transplantation [152,153,154]. In the majority of cases, PTLD is associated with active replication of EBV after either primary infection or reactivation during treatment with immunosuppressive drugs. EBV^+^ PTLDs express the latency III program (see Section 2), in which the six viral latent genes are expressed, similar to LCLs.

## 6. Burkitt Lymphoma

Burkitt lymphoma (BL) is a very aggressive B cell non-Hodgkin lymphoma, characterized by its fast growth rate. BL was the first tumor associated with a virus. In 1964, Michael Anthony Epstein, Yvonne Barr, and Bert Achong identified viral particles in a lymphoma recurrently suffered by African children, the so-called Epstein–Barr virus (EBV) [1]. BL is one of the most prevalent cancers in children from the African equatorial belt, accounting for up to 74% of childhood malignancies [155,156].

The fifth edition of the WHO classification regarding hematolymphoid tumors currently distinguishes between EBV-positive BL (EBV^+^ BL) and EBV-negative BL (EBV^−^ BL), since they form discrete biological groups based on their molecular features and thus use the virology rather than the epidemiology as the criteria to classify the BL [142]. EBV infection confers BL cells mechanisms to evade apoptosis, an essential feature during the onset of this disease [157,158,159,160,161]. EBV^−^ BL are considered “mutational driven” while EBV^+^ BL are considered “virus driven”. The main differences between both subtypes highlighted in the current WHO classification are the following: EBV^+^ BL harbors higher somatic hypermutation levels, fewer driver mutations, and lower frequency of mutations in specific genes (see below) [162]. These differences between EBV^+^ BL and EBV^−^ BL are summarized in Table 1. However, it is worth mentioning that, for more than 50 years, BL has been classified into three subgroups depending on the epidemiological context and geographical location: endemic BL (eBL), sporadic BL (sBL), and immunodeficient-associated BL (iBL) [155,163]. The association of BL with EBV infection varies depending on geographical distribution and associated risk factors, accounting for 98% of BL in Africa (formerly known as eBL), 30–40% of the BL cases associated with immunodeficiencies (formerly known as iBL), and 5–10% of the rest of BL cases (formerly known as sBL). The eBL variant strikingly correlates with regions where malaria is endemic and with early EBV infection events, mainly Equatorial Africa and Papua New Guinea. This evidence indicates a strong association of malaria infection with EBV and BL pathogenesis. The peak of incidence is found at the age of 6 and it is more common in boys than in girls [164].

EBV^−^ BL (sBL) is mainly found in North America, Europe, and some regions of Asia. Its incidence is low: two cases per million population <18 years old. In some regions of South America, southern Europe, North Africa, and the Middle East, there is an intermediate association of BL with EBV. Interestingly, overall EBV prevalence in patients with BL has decreased from 64% in 1969–82 to 54% in 2009–2021) [165]. The so-called iBL variant is mainly related to HIV infection, although this association is not as strong as the one with malaria. HIV-BL is more common in those patients who still show an intermediate CD4 T-cell response and its incidence is very variable [166,167]. BL is also found in organ transplant recipients. 

The major common feature shared by all BLs (both EBV^+^ and EBV^−^ BL) consists of a translocation involving chromosome 8 [168], where the oncogenic transcription factor *MYC* locus is located [169], and most frequently chromosome 14 at the immunoglobulin heavy chain locus or, less commonly, chromosomes 2 or 22 at the immunoglobulin light chain loci (this will be discussed in the following sections). The molecular mechanisms leading to MYC translocation in BL remain largely unknown. Breakpoints in the 8;14 translocation affect the S (switch) regions of the heavy chain loci, suggesting that it originates from aberrant class switch recombination (reviewed in [170]). Also, it has been recently reported that EBV reactivation in lymphoblastoid cell lines induces *MYC* and *IGH* spatial proximity, which would facilitate the t(8;14) translocation [171]. The association between EBV and BL was reported after very early EBV’s discovery. A study from 1978 that performed prospective serological investigation in a collection of blood samples of children from Uganda, where BL is endemic, showed that those who had developed BL displayed statistically significantly higher titers of antibodies against EBV viral capsid antigens compared to healthy children.

Interestingly, no differences were found in antibody titers against early antigens or nuclear antigens. These higher antibody titers were associated with a higher risk of developing BL [172,173]. The correlation between high antiviral-capsid antibody titers and BL was also reported for sporadic BL later on.

Together with EBV, there are other risk cofactors that contribute to the development of BL, some of which depend on the geographical location or genetic predispositions, and they vary depending on the BL subtype. Among them, chronic immune activation due to malaria or HIV infection is considered a high risk of BL development, especially malaria in eBL [174]. Repeated infections of *Plasmodium falciparum* (the parasite that causes malaria) during childhood in malaria-endemic regions together with the high incidence of EBV infection are directly associated with BL development. Chronic immune activation due to malaria weakens immune control over EBV and promotes B cell proliferation and EBV reactivation [175]. This environment of rapid B cell proliferation and increased germinal center formation increases the likelihood of oncogenic events, especially MYC translocations. The more the germinal center reactions, the higher the activation-induced cytidine deaminase (AID) activity. In the germinal center, AID is responsible for the somatic hypermutations (SHM) and class switch recombination (CSR) events needed for proper antibody diversification [176,177]. In BL, increased AID activity due to different factors, including chronic immune activation, leads to off-target breaks or errors, making *IG::MYC* translocations more likely [178]. Similar to malaria, the dysregulation of the immune system caused by HIV [179] increases the risk of developing BL (HIV^+^ individuals are at 10–100 times higher risk of developing BL compared to HIV^−^ individuals). HIV-BL is more similar to sBL in terms of clinical and pathological features [155,180]. The mycotoxin aflatoxin B1 is a food contaminant more common in the African regions with higher EBV prevalence, and it has been shown to stimulate EBV-mediated transformation [181]. Recently, the secretion of the cytokine CCLC2 [182] and the downregulation of the TGB1 gene [183] have been shown as possible mechanisms to explain the aflatoxin B1 role in BL.

**Table 1:** Major differences between EBV^+^ and EBV^−^ BL. Modified from [155,156,180].

Current WHO Classification	EBV^+^ BL	EBV^−^ BL
BL subtype (traditional classification)	95% eBL 5–10% sBL 30–40% iBL	90–95% sBL 60–70% iBL
Geographical distribution	Equatorial belt of Africa and Papua New Guinea (eBL)	Worldwide (>North America, northern Europe and east Asia)
Age	Children Median age, 6 y/o (eBL)	Adults All ages
Presentation site	Jaw, eye, abdomen, kidneys and ovaries (eBL) Abdomen, lymph nodes and bone marrow (sBL)	Abdomen, lymph nodes and bone marrow
Annual incidence	40–50 per million children younger than 18 years. 50% of all childhood cancers and up to 90% of lymphoma Twice more frequent in boys than girls	2–3 per million 3–5 times more frequent in boys than girls
Cofactors	Malaria infection and aflatoxin B1 in eBL, HIV infection in iBL	
Precursor cell	Memory and germinal center B cell	Germinal center B cell
Prevalent Ig breakpoint	VDJ region, switch (s)μ in some cases in eBL Sμ, Sα or J region in sBL Sμ in HIV-associated BL	Sμ, Sα or J region in sBL Sμ in HIV-associated BL
Prevalent MYC breakpoint	>100 bp upstream of first exon in eBL Between exons 1 and 2 or in 5′ near the first exon in sBL and iBL	Between exons 1 and 2 or in 5′ near the first exon
Mechanism of BL pathogenesis	Virus-driven	Mutational
Mutational landscape	++ AID activity Higher levels SHM	More driver mutations (apoptotic pathway)

There are different hypotheses regarding the origin of BL. Whether it originates from germinal center B cells or mature memory B cells is unclear. Both sporadic and endemic BL present somatic mutations in the rearranged V region genes, meaning that they derive from either germinal center or germinal center descendants [184,185]. While the surface phenotype of BL resembles that of germinal center cells, the Ig features support the hypothesis that BL is a tumor arising from latently infected memory B cells. It has also been postulated that the cell of origin could be a memory B cell that re-enters the germinal center and that there may be differences depending on whether the BL is EBV^+^ or EBV^−^ [185]. In any case, the germinal center reaction is essential for the pathogenesis of this disease, since it is where key molecular events, such as *MYC* translocations, take place [177,178]. 

The interaction of EBV with B cells takes place through a gp350/220 EBV glycoprotein binding to CR2 in the surface of B cells [44,53]. It is worth mentioning that naïve B cells show the highest CR2 (and CR1) expression compared with germinal center and mature B cells [186], which leads to the idea that naïve B cells are the cells most likely to be infected by EBV, since EBV infection rates directly correlate with CR2 expression levels [187]. Of note, *CR2* has been reported as a direct MYC target gene, demonstrating that modulation of MYC expression directly affects the EBV loads found in the surface of B cells [188]. Thus, *MYC* translocations in BL occurring in germinal center cells would increase the density of CR2 in the plasma membrane of germinal center cells increasing the likelihood of EBV infection. Although this has not been proven, CR2 upregulation in germinal center cells by deregulated MYC is in line with the hypothesis in which *MYC* translocations favor EBV infection that will be discussed in the following sections.

Most BL cells display the Latency I program in which only EBNA1, EBERs, and BARTs are expressed (Figure 2) [156]. It is unclear whether BL originates from either B cells in Latency I or a specific clone of B cells in Latency III that switches to a Latency I program [189]. The latency I program of BL cells suggests that the B cells leaving the germinal center cannot completely differentiate into resting mature B cells since they keep proliferating (cannot transit to latency 0), most likely due to MYC deregulation [189,190]. *EBNA1* is mainly expressed from Qp, wherein Wp and Cp promoters are shut down. However, there are cases in which a different latency state of BL cells is found, where *EBNA1* is transcribed from Wp instead of Qp, together with *EBNA3A/B/C* and *EBNA-LP*. This is known as “Wp-restricted” BL [189]. These cells are infected with an EBNA2-deficient EBV in which neither EBNA2 nor LMP1 are expressed. Moreover, single-cell analysis of BL samples also revealed another form of latency in which EBNA2 but not LMP1 is expressed. This suggests that LMP1 or EBNA2-induced *LMP1* is not compatible with *MYC* translocation in BL. All three latency subtypes seem to be related with their ability to protect BL cells from the apoptosis induced by MYC, wherein the latency I is the most sensitive, Wp restriction is the most resistant, and EBNA2+/LMP1- shows an intermediate phenotype [191].

Several studies have focused their attention on deciphering the mutational landscape of BL, especially the differences between EBV^+^ and EBV^−^ BL [42,162,192,193]. While the two entities cannot be distinguished only on basis of the mutational profiling, mutations in specific genes have been reported to differentially occur in EBV^+^ vs. EBV^−^ BLs, especially in genes involved in BCR signaling, proliferation, TP53 pathway, chromatin remodeling, and sphingosine-1-phosphate signaling (reviewed in [163]). *MYC* is also frequently mutated (see Section 9). At the genomic level, EBV^+^ BL shows higher mutational burden but fewer driving mutations than EBV^−^ BL, especially in those genes involved in apoptosis [162]. One of the most striking differences is found in the TCF3 pathway, as EBV^−^ BL has been reported to harbor higher mutational rates in the *TCF3* gene and its negative regulator *ID3* (activating and inactivating mutations, respectively), leading to increased TCF3 pathway activity [42,162,192]. Mutations in genes involved in epigenetic regulation processes were found significantly more frequently in EBV^+^ BL compared with EBV^−^ BL [162].

Transcriptomic studies have also revealed that EBV^−^ BL shows higher levels of *MYC*, *DDX3X*, *CCND3,* and *TP53* mRNA compared to EBV^+^ BL. Also, it has been recently reported that the expression of SOX11 (SRY-box transcription factor 11) is expressed only in EBV^−^ BL, although its biological significance is still unknown [194]. On the other hand, AID activity has been recurrently reported to be much higher in EBV^+^ BL, in line with the increased mutational rate of the AICDA gene compared with EBV^−^ BL [162]. Interestingly, EBV^+^ BL is characterized by increased PI3K activity via downregulation of PTEN, followed by mTORC1 activation [192]. 

LCLs generated by EBV infection show a global genome demethylation as compared to normal lymphocytes, but this has not been demonstrated in BL cells [195]. In contrast, in BL, NPC, and EBVaGC there is a tendency toward CpG island hypermethylation [196]. It has been shown that methylation changes in the promoters are responsible for the differential expression of genes between EBV^+^ and EBV^−^ BL. For example, ID3 promoter methylation is higher and its expression is lower in BL EBV^+^ BL than in EBV^−^ BL [197]. Several viral genes have functions linked to epigenetic regulation mediated by EBV, such as the recruitment of polycomb repressive complex 2 (PRC2) by EBNA3A, the recruitment of HDAC1/2 by EBNA3C, or the interaction of the SNF-SWI remodeler complex with EBNA2, but none of them are expressed in the genetic program latency I of BL (reviewed in [20,196]).

## 7. EBV Genes That Contribute to Lymphoma Progression

As already discussed above, latency I is the main latency program in BL, together with a minor percentage of cells showing the so-called “Wp-restricted” or the LMP1^−^/EBNA2^+^ expression profiles. We will focus on the oncogenic activities driven by the EBV genes expressed in BL. However, an effect of the rest of the latent proteins expressed only during the early process of the primary infection cannot be ruled out as an important event in the BL onset. This is the case of LMP1 and LMP2 membrane proteins, whose expression is restricted to latency III/II programs and thus not expressed in BL cells. LMP1 and LMP2 have the ability to mimic CD40 [198] and BCR [199] signaling, respectively, likely ensuring EBV-infected B cell survival during the germinal center reaction. The EBV genes more linked to B cell transformation are briefly reviewed below.

EBNA1: This gene encodes a transcription factor that binds both viral and host promoters and it is required for proper EBV episome segregation [200]. EBNA1 is the only gene expressed in all latency and lytic programs (except for latency 0) and thus the major latent gene expressed in BL. The potential oncogenic capacity of EBNA1 has been a matter of extensive study and debate throughout the last decades. In fact, EBV lacking EBNA1 is much less efficient in the immortalization of B cells than wild-type EBV [201]. Reports using transgenic mice claim that EBNA1 does not promote lymphomagenesis [202], although EBNA1 has been reported to induce B cell neoplasia in mice [203], in an MDM2-dependent manner [204]. EBNA1 inhibition increases cell death in BL cells; thus, it seems to have a pro-survival role in BL [205]. It has been reported that EBNA1 impairs p53 activity through different mechanisms [206] and that the EBNA1-VAV1 interaction impairs BIM expression [207], altogether conferring resistance to apoptosis in BL.

EBNA2: This encoded transcription factor, which does not directly bind to DNA, is essential for the transformation of B cells in vitro by EBV [208], while the rest of viral latent genes are dispensable [209]. EBNA2 has been reported to functionally replace intracellular Notch signaling [210], supporting cell survival and lymphomagenesis in BL. The MYC oncogene and LMP1 are directly activated by EBNA2 [211].

EBNA3: The EBNA3 family of transcription factors is composed of three isoforms: EBNA3A, EBNA3B, and EBNA3C. Among them, EBNA3A and EBNA3C are essential for B cell transformation, while EBNA3B is not [212]. In fact, EBNA3B has been proposed to have a tumor suppressive role, since its inactivation drives lymphomagenesis and immune evasion [19]. EBNA3A and EBNA3C confer survival advantage by blocking apoptosis through the inhibition of BIM [213]. This is interesting since MYC overexpression can induce apoptosis via BIM [214]. Finally, EBNA3A regulates MCL-1 mitochondrial organization through the upregulation of BLF-1 expression [215], impairing apoptosis. This would be an EBV-mediated survival mechanism that resembles the MCL-1 function during germinal center in mice [216]. 

BHRF1: This gene encodes for an early lytic gene thought to be only expressed during lytic replication from Wp. However, its expression has been detected in other latency programs and more importantly in “Wp-restricted” BL. As a BCL2 homologue, BHRF1 interferes with BIM and other proapoptotic proteins in BL cells, conferring resistance to apoptosis [101,158,217] and contributing to lymphomagenesis.

## 8. MYC Protein and Biology

**Figure 4:**
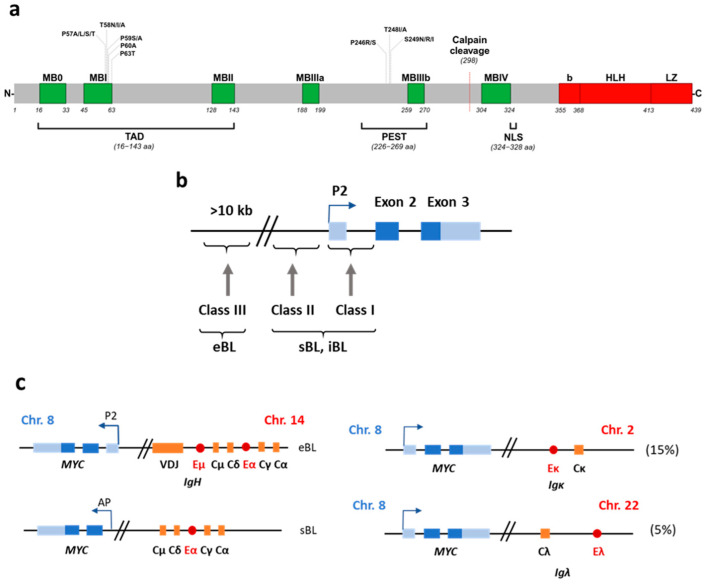
The oncogenic MYC transcription factor and its translocations within the different Ig loci found in Burkitt lymphoma. (**a**) Scheme of the MYC protein. The conserved boxes among MYC proteins are in green. The bHLHLZ domain is in red. The most prevalent mutations in BL are indicated. TAD, transactivation domain; PEST, degradation domain; NLS, nuclear localization signals. (**b**). Schematic representation (not to scale) of the breakpoints in the chromosome 8 in BL. The three exons of the MYC genes are the blue boxes, with the coding regions in darker blue. The major promoter, P2, is shown. The three classes of breakpoints with the chromosome 14 (immunoglobulin heavy chain genes) are indicated as class I, II, and III and the BL subtype where it is more prevalent. The breakpoint in the eBL typically occurs > 10 kb upstream of the first *MYC* exon and in the first noncoding exon or first intron in the sBL. (**c**) Schematic figures (not to scale) of prototypical translocations MYC::IGH to heavy chain genes (left schemes, 80% of the BL cases), MYC::IGK to light chain kappa genes (upper right scheme, 15%), and MYC::IGL light chain lambda genes (lower right scheme, 5%). In the t(8;14) translocation, the break points are the switch regions of the constant gene segments encoding the IgH isotypes and are upstream of *MYC*. In eBL, the breakpoint in the chromosome 14 is more frequently in the J segment. The predominant breakpoint in sBL occurs in the Cµ (M) regions of the H chain locus. In the t(2;8) and t(8;22) translocations, the kappa and lambda light chain lie far downstream of *MYC*. AP refers to an alternative promoter used in this type of translocations. The enhancers are denoted as red circles. Adapted from [163,170]. Partially Created in BioRender. Garcia, L. (2024) BioRender.com/y04j261.

MYC is the prototypical transcription factor of the superfamily of factors that contain the helix-loop-helix-leucine zipper domain (HLH-LZ). MYC (also called c-MYC) is one of the three members of the MYC family. The other two proteins of the family are MYCN (also called N-MYC) and MYCL1 (also called L-MYC). All paralogs share the HLH-LZ domain as well as the other four conserved regions of the molecule, called MYC boxes (Figure 4a). However, MYC is by far the member of this family more heavily involved in cancer and is also the most ubiquitously expressed in human tissues. 

All *MYC* genes operate as transcription factors forming heterodimers with MAX, also an HLH-LZ protein [218]. The MYC-MAX heterodimer is the active form, which binds to specific DNA sequences called E-boxes (canonical sequence CACGTG) in the regulatory regions of target genes. The MYC network includes other components of the HLH-LZ family such as the MXDs and MLX with different functions in gene expression regulation upon binding to E-boxes in the DNA [219,220]. 

Transcriptomic analyses revealed that MYC can regulate thousands of genes, when comparing cells with low vs. high MYC expression. However, the number of regulated genes depends on the MYC levels in a particular cell type. The number of MYC-binding sites revealed by genome-wide technologies ranks between 7000 and 15,000 in different models and regulates more than 1000 genes [221,222,223,224,225]. Indeed, MYC is bound at one or more sites of the regulatory regions of 10–15% of human genes [225]. In agreement with the large number of MYC target genes, the overexpression of MYC deregulates a series of biological functions such as cell cycle progression, nucleotide biosynthesis, energy metabolism, protein synthesis and ribosome genesis, genomic maintenance, immortalization, and differentiation block [226,227,228]. These series of acquired or enhanced functions in MYC-overexpressing cells would confer a competitive advantage triggering the MYC-mediated oncogenesis. Not surprisingly, *MYC* is dysregulated in more than 50% of human malignancies of all types [229,230], although it is particularly prevalent in lymphoma and leukemia [231,232].

## 9. MYC Deregulation in BL

MYC was the first oncogenic transcription factor identified, initially as the transforming genes of an avian retrovirus that induced several tumors, most prominently, myelocytomas [233,234,235]. Interestingly, the original virus did not induce lymphoma, although when later isolated, it induced T and B lymphomas [236]. In the 1970s, a recurrent translocation between the chromosomes 8 and 14 was described in BL [237]. In 1982, it was reported that the translocation involved the transforming sequences of the myelocytomatosis (MYC) retrovirus MC29, being the first consistent cancer-associated translocation described [169].

Virtually all BL carry a reciprocal translocation that places the *MYC* gene (in chromosome 8) in the vicinity of immunoglobulin regulatory regions. MYC plays a prominent role in germinal center formation [238,239]. In the germinal center, the activation-induced cytidine deaminase (AID) initiates somatic hypermutation and class switch recombination [240], but through a defective process, it can also induce Ig/MYC translocations [178,241].

The most common translocation in BL and other lymphomas is the t(8;14) where *MYC* is placed in the IgH region (80% of the BL cases) but it can also be translocated to the light chin Ig genes: the κ light chain in the t(2;8)(p11;q24) translocation (15% of the cases) and the λ light chain in the t(8;22)(q24;q11) translocation (5% of the cases) [242]. In the most common t(8;14) translocation, the breakpoint is upstream of the MYC gene and in the other two types, it is downstream (Figure 4b). Interestingly, there is a difference in the translocation point depending on the EBV association. In EBV^−^ sBL and HIV-BL, the breakpoint of the t(8;14) translocation in the *MYC* gene occurs usually between exons 1 and 2 of *MYC* (being exon 1 non-coding) or upstream in the proximity of the first exon. In the EBV^+^ eBL, the breakpoint usually occurs far upstream of the *MYC* gene, in some cases more than 100 kb, and most often affects the VDJ region or the M (Cμ) region in some cases (reviewed in [170,242]) (Figure 4c). It is of notable that ~2% of BL carry *MYC* translocations not affecting the immunoglobulin genes [243].

The leukemic form of BL is the acute lymphoblastic leukemia type L3, which also carries the *MYC::IGH* translocation [244]. However, *MYC* translocation is also frequent in other B cell malignancies, more prominently in diffuse large B cell lymphoma (70%), mantle cell lymphoma (14–20%) [232,245,246], and multiple myeloma (15–50%) [247].

This translocation results in *MYC* expression under the control of immunoglobulin gene regulatory elements, including enhancers, which are very active in mature B cells. Thus, the translocation leads to the overexpression of *MYC* or to the loss of the physiological regulation of MYC. Interestingly, the normal *MYC* allele is transcriptionally silent in BL [248,249] and thus most of the MYC protein in most BL cells is derived from the translocated allele. Whereas *MYC* translocation is rare in solid tumors, *MYC* is amplified or transcriptionally deregulated in many tumors of virtually any type. Overall, the reported incidence of *MYC* deregulation through translocation, amplification, transcriptional deregulation, or mutation ranges from 50% to 70% [229,230].

Besides *MYC* translocations, 30–50% of BL carry mutations within the *MYC* gene [193,250,251,252]. T58 is one of the most prevalent mutated codons (Figure 4a). Phosphorylation of this threonine marks MYC for ubiquitylation and degradation [253], resulting in a more stable MYC protein and higher MYC levels in the cell. Other genes have been found to be recurrently mutated in BL, such as ID3, p53, GNA13, RET, PIK3R1, ARID1A, and SMARCA4 [193,252]. Interestingly, ID3 mutations are much more frequent in BL (30% of the cases) than in any other high-grade lymphoma such as DLBCL [252].

The first animal model generated for MYC-driven cancer was the *Eμ-Myc* transgenic mouse, in which MYC expression is targeted to the lymphoid compartment by the immunoglobulin heavy chain gene promoter and enhancer. These mice develop cell lymphomas [254,255] that do not faithfully reproduce the BL characteristics, as the tumor cells are at the pre-B developmental stage whereas BL is composed of more mature B cells. However, these transgenic mice demonstrated the relevance of *MYC* deregulation in B cell malignancies. Later on, additional transgenic mice lines have been generated that better reproduce human BL: (i) a mouse model carrying a single copy of the 240-kb IgH/c-*Myc* translocation region [256], (ii) a model that carries the murine *Myc* cDNA inserted in the *IgH* locus in a site that corresponds to the human t(8;14) translocation break [257], (iii) mice with MYC linked to the 3′ *IgH* locus control region (3′ LCR) [258], or (iv) mice with combined MYC overexpression that constitutive activation of the PI3K [259].

## 10. Roles of MYC in the Pathogenesis of BL

The role of EBV in BL pathogenesis has been a matter of debate. The canonical mechanism postulates that EBV infection triggers the malignant transformation of B cells in the germinal center of the lymph nodes. However, there is no doubt regarding the pivotal role of *MYC* translocations in BL being the hallmark of this disease and probably the main driver of BL. What happens first, whether it is *MYC* translocations or EBV infection during the onset of BL is not clear. The “*virus first*” is the most accepted hypothesis, at least for the endemic form affecting children in Africa and other malaria-exposed countries. The following data support this hypothesis:

(a) EBV antibodies in serum precede the onset of BL in African children. Although the original data are based on a limited number of cases, this is likely the most compelling argument. Indeed, the WHO declared EBV as the causing agent of BL [260];
(b) The analysis of clonality based on the number of terminal repeats of the EBV genome in BL cell lines suggests that the tumors arise clonally after infection by EBV [261];
(c) EBV transforms resting B cells to generate lymphoblastoid cell lines. These are immortalized B or semi-immortalized cell lines or LCLs. These cells are not fully immortal but can grow for a large number of population doublings retaining a diploid karyotype without becoming tumorigenic [256];
(d) EBV could induce *MYC* gene translocation via activation of AID. The viral EBNA3C protein induces AID expression, which could generate aberrant chromosomal translocations including those that involve *MYC* [178,241,262];

However, other evidence argues against this “*virus first*” model, such as the following:

(a) First, in most sporadic BL cases, EBV infection is absent while *MYC* is translocated in all cases. Moreover, although there are differences in the prevalence of some genetic alterations and gene expression patterns, there are no differential molecular markers in the mutational landscape between EBV^+^ and EBV^−^ lymphomas, including mutations in the *MYC* gene [42,162,192,250,263];
(b) More than 90% of the human adult population has been infected by EBV, but oncogenic events related to EBV infection have a very low incidence, and in fact, the prevalence of EBV^+^ BL is decreasing [165];
(c) Remarkably, *MYC* translocation is not found in other tumors associated with EBV infection, encompassing nasopharyngeal cancer (where EBV association is above 90%) [109], gastric cancer, and Hodgkin lymphoma [264,265,266];
(d) The EBV genes required for B cell transformation into lymphoblastoid cell lines are not expressed in BL. Indeed, only the latent EBNA1 protein is consistently expressed in BL (see Section 2). EBNA1 is required for viral DNA replication but has no transforming effects, and it is unable to transform B cells. Moreover, MYC represses the EBV-transforming *LMP1* gene [267]. It has been reported that EBNA2 and EBNA3C induce *MYC* expression [211,268,269]. However, neither EBNA2 nor EBNA3C are expressed in most BL. Moreover, a relevant fraction of BLs (15%) carries a deletion of the *EBNA2* gene in the viral genome [189];
(e) EBV-generated lymphoblastoid cell lines express some viral oncogenes (EBNA2, LMP1) but do not carry *MYC* translocations [270];
(f) The receptor for EBV in B cells, *CR2*, is a direct MYC target gene. Thus, B cells expressing high MYC levels as a result of the translocation will also express higher CR2 levels and thus will be more efficiently infected by EBV [188].

**Figure 5:**
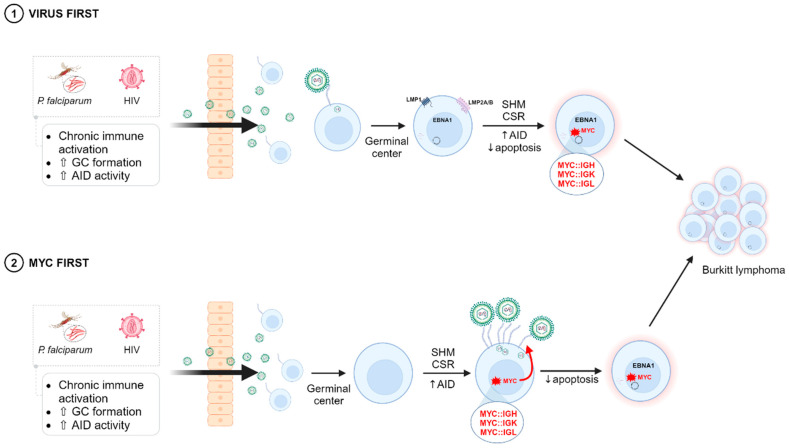
Two alternative models of EBV infection and *MYC* translocation in B cells prior to Burkitt lymphoma development. Chronic immune activation triggered by malaria or HIV leads to increased B cell proliferation, germinal center formation, and AID activity. In the *virus first* model, higher loads of EBV due to the weakened immune system favor EBV-mediated infection of B cells, leading to the increased susceptibility of MYC translocations during somatic hypermutation (SHM) and class switch recombination (CSR) in the germinal center. In the *MYC first* model, the chronic immune activation due to malaria or HIV makes the B cells prone to MYC translocations due to errors during SHM or CSR. Translocated *MYC* would lead to higher CR2 density on the surface of B cells, increasing the probability of EBV infection. In both models, EBV would confer the resistance mechanisms to apoptosis needed for the B cell to cope with the deregulated MYC activity, leading to Burkitt lymphoma development. Created in BioRender. Garcia, L. (2024) BioRender.com/y04j261.

The “MYC first” hypothesis has been proposed earlier [270,271] and the MYC-mediated up-regulation of the CR2 receptor gives further support to this hypothesis. According to this mechanism, *MYC* translocation (and thus *MYC* deregulation) will occur stochastically at one or a few cells that will express more CR2 in its membrane, which in turn will lead to a more efficient infection. The “MYC first” mechanism would be compatible with the epidemiological data and the viral clonality data, as the subsequent EBV infection will co-adjuvant to lymphoma development. Figure 5 schematizes both mechanisms to explain the EBV-BL association.

It must be noted that the *virus fist* or *MYC first* mechanisms are not mutually exclusive. So, it is possible that one BL arises after the infection, and the virus would use the CR2 expressed at basal levels in the germinal center B cell. Although BL does not express EBV-transforming genes (e.g., EBNA2, LMP1), a “hit and run mechanism” by which these genes or the translocation-promoting EBNA3C is expressed after infection contributes to B cell proliferation and then becomes silenced in the growing lymphoma, cannot be ruled out. This may increase the pool of cells increasing the chance of *MYC* translocation. However, in other tumors, *MYC* translocation may come first, and the virus will find cells with a higher density of receptors in the membrane, which will be preferentially infected. Actually, at least one case of BL has been reported in which the EBV^+^ cells and EBV^−^ cells coexist in the same tumor, despite the fact that both cells show the same clonal origin [272].

Regardless of whether EBV is or is not the primary cause of BL, it is clearly a risk factor contributing to carcinogenesis even if the tumor is initiated by a *MYC* translocation event. A number of EBV-mediated mechanisms are described.

EBV confers resistance to apoptosis as described above (Section 6) and contributes to the metabolic reprogramming of B cells [273]. At least two viral genes have proven oncogenic properties (*EBNA2* and *LMP1*). Moreover, it has been recently shown that the latent EBV infection collaborates with Myc over-expression to induce BL-like lymphomas in mice [274].

EBV contributes to the scape from the immune surveillance of BL cells. This might also be the mechanism for the higher BL incidence in areas where malaria is endemic and for the HIV-associated BL.

Interestingly, lymphoblastoid cells (immortalized by EBV) are not tumorigenic in nude mice but when engineered to constitutively express a *MYC* gene, the cells become tumorigenic [275]. It is noteworthy that MYC represses the expression of EBV lytic genes by interacting with the EBV genome origin of lytic replication, preventing the expression of *BZLF1* and therefore the lytic cascade of gene expression [276]. Therefore, MYC would enhance viral infection on one hand but prevent the lytic cycle of infected cells on the other hand, thus contributing to the latency I program typical of BL cells.

## 11. Concluding Remarks

It is undisputed that the MYC oncogene, by chromosomal translocation to the immunoglobulin genes, is the hallmark and the cause for BL development. It is also established that a relevant fraction of BL is EBV^+^. However, the molecular mechanisms that may explain the connection between EBV infection and *MYC* translocation are not yet clear. A role of MYC in BL pathogenesis is supported for the ample repertoire of biological effects elicited by MYC that impinge on cell proliferation. Indeed, MYC is deregulated in a majority of human cancers including lymphoma, and it is usually associated with a poorer outcome. However, in contrast to non-lymphoid tumors where translocations are rare, *MYC* is frequently translocated and deregulated in B cell lymphomas and in virtually all cases of Burkitt lymphoma. It is also clear that BL is associated to EBV infection in a relevant fraction of cases. This association reaches the 95% of the cases in the so-called endemic variant of the disease that occurs in geographical areas were malaria is prevalent, as equatorial Africa, whereas in other areas, the extent of this association is much less frequent. A debate regarding whether EBV is sufficient to trigger BL or if it is a risk factor exists. Some data support the most accepted mechanism, which is that viral infection of B cells in the lymph nodes is the triggering event that leads to *MYC* translocation followed by lymphomagenesis. However, other data argue about this “*virus first*” hypothesis and propose the “*MYC first*” hypothesis by which the *MYC* translocation is the first event and the antiapoptotic effect of EBV will critically favor lymphomagenesis. Moreover, the fact that MYC induces the expression of the virus receptor, CR2/CD21, will also favor the EBV infection in B cells already carrying the *MYC* translocation.
